# Effects of candesartan on cerebral microvascular function in mild cognitive impairment: Results of two clinical trials

**DOI:** 10.1177/17474930231153313

**Published:** 2023-01-30

**Authors:** Brandon Henley, Maureen Okafor, Ambar Kulshreshtha, Antoine Trammell, Ihab Hajjar

**Affiliations:** 1 Department of Neurology, Emory University School of Medicine, Atlanta, GA, USA; 2 Department of Family and Preventive Medicine, Emory University School of Medicine, Atlanta, GA, USA; 3 Department of Medicine, Emory University School of Medicine, Atlanta, GA, USA; 4 Department of Neurology, University of Texas Southwestern Medical Center, Dallas, TX, USA

**Keywords:** Candesartan, cerebrovascular reactivity, MRI, mild cognitive impairment

## Abstract

**Background:**

Cerebral microvascular dysfunction is commonly seen in Alzheimer’s disease (AD) and vascular cognitive impairment (VCI). Cerebrovascular reactivity (CVR) to CO_2_ reflects cerebral microvascular health and may be modulated by the renin–angiotensin system (RAS). This study aimed to investigate the effects of RAS modulation on CVR in individuals with mild cognitive impairment (MCI) due to underlying vascular or AD etiologies.

**Methods:**

This study presents findings of candesartan’s effects on the secondary outcomes of two double-blind randomized clinical trials of 12-month therapy of candesartan versus lisinopril in VCI (CALIBREX (Candesartan vs Lisinopril Effects on the Brain and Endothelial Function in Executive MCI)) and candesartan versus placebo in prodromal AD (Candesartan’s Effects on Alzheimer’s Disease and Related Biomarkers (CEDAR)). Primary outcome results of these trials have been reported in previous publications. Participants underwent identical brain blood oxygenation level dependent (BOLD)-CVR in response to a 2-min CO_2_ challenge at baseline and 12 months. Regions of interest and voxel-wise CVR maps were derived from BOLD signal changes during CO_2_ challenge. CVR effects were compared between candesartan and lisinopril (CALIBREX) and candesartan and placebo (CEDAR) using mixed-model repeated measures.

**Results:**

Data from 102 participants in the CALIBREX study (mean age = 65 years, 45% female, 63% African American) and 59 in the CEDAR study (mean age = 67 years, 32% female, 20% African American) were analyzed. Candesartan was associated with improved whole brain CVR compared to placebo in the CEDAR study (adjusted within-group mean difference for candesartan = 0.27 (95% confidence interval (CI) = 0.006, 0.53) vs placebo = −0.17 (95% CI = 0.42, 0.08), p-value = 0.018), and compared to lisinopril in the CALIBREX study (adjusted within-group mean difference for candesartan = 0.28 (95% CI = 0.10, 0.46) vs lisinopril = −0.08 (95% CI = −0.31, 0.14), p-value = 0.012), independent of blood pressure. In an exploratory meta-analysis of the two trials, improved CVR in the hippocampus was linked to improved attention and working memory (p = 0.044) and a trend for improved executive function (p = 0.087) with candesartan therapy.

**Conclusion:**

This study suggests that candesartan is associated with improved microvascular function in MCI, and these findings are independent of its blood pressure effect in these VCI and prodromal AD populations.

## Introduction

Mild cognitive impairment (MCI) is highly prevalent in older adults and expected to increase significantly with the aging global population.^1,2^ The two most common causes are Alzheimer’s disease (AD) and vascular cognitive impairment (VCI).^2^ Microvascular dysfunction in the cerebral circulation is common underlying mechanisms for both diseases as reflected by cerebrovascular reactivity (CVR) to increase in end-tidal carbon dioxide (CO_2_).^3–5^ Although traditionally measured using Trans-cranial Doppler technique, CVR during brain magnetic resonance imaging (MRI) is becoming more accessible.^6,7^ Patients with either MCI due to AD (termed prodromal AD) or VCI have significant microvascular dysfunction reflected by lower CVR values relative to cognitively unimpaired individuals.^3,8^ Hence, targeting microvascular function may provide significant therapeutic benefits to MCI, independent of the underlying vascular or AD etiologies.

Our work has focused on the renin–angiotensin system (RAS) due to its significant influence on microvascular health. RAS can be modulated by angiotensin-converting enzyme inhibitors (ACEIs) or angiotensin receptor blockers (ARBs). Independent of their effects on blood pressure, ACEI and ARB may also improve cerebrovascular function via other mechanisms, including neurohumoral modulation, anti-inflammatory mechanisms, and bradykinin-mediated vasodilation.^9,10^ We have previously reported that candesartan, an ARB, may have unique and superior effects on cognition relative to other antihypertensive medications including lisinopril, an ACEI.^11,12^ RAS has the potential of influencing cerebral microvascular function.^12,13^

### Aim and hypothesis

In this study, we aimed to compare the effects of 1-year treatment of candesartan on CVR in these two independent populations of MCI: (a) VCI and (b) prodromal AD. We hypothesized that candesartan has superior effects on CVR compared to placebo or lisinopril in these MCI populations with underlying vascular or AD etiologies.

## Methods

### Study design

**Figure 1.**
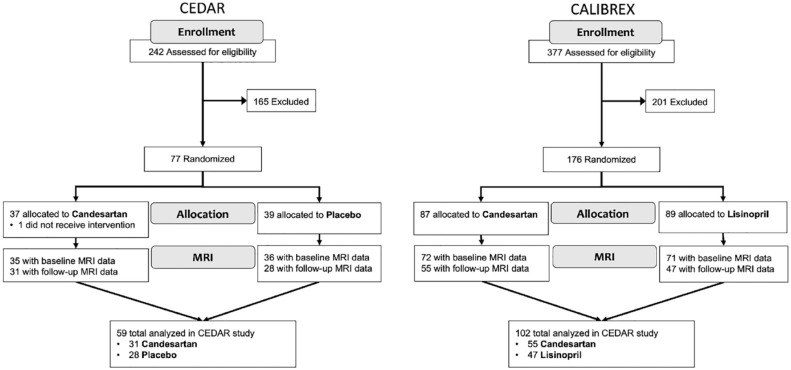
CONSORT diagram of CEDAR and CALIBREX clinical trials.

CALIBREX (Candesartan vs Lisinopril Effects on the Brain and Endothelial Function in Executive MCI) and CEDAR (Candesartan’s Effects on Alzheimer’s Disease and Related Biomarkers) clinical trials were randomized, double-blind studies conducted in Atlanta, Georgia. Both trials compared the effects of 12-month oral candesartan treatment with lisinopril (CALIBREX) or placebo (CEDAR) on the brain and followed the Consolidated Standard of Reporting Trials (CONSORT) guidelines (Figure 1). All participants were blinded to their randomized group assignments.^11^ The trials were reviewed and approved by the Emory Institutional Review Board, and screened participants provided written informed consent. Results of the primary outcomes of these two trials have been described in previous publications^11,14^ and can be found on ClinicalTrials.gov (NCT01984164 and NCT02646982). This study presents findings of candesartan’s effects on secondary outcomes of these two clinical trials.

### Participant eligibility

The CALIBREX trial recruited individuals aged ⩾55 years with hypertension and executive MCI as an indicator of VCI. Hypertension was defined as systolic blood pressure (SBP) ⩾140 mm Hg or diastolic blood pressure (DBP) ⩾90 mm Hg, or receiving antihypertensive medication. Those who were on antihypertensive medication were guided through a drug taper and washout period before commencing the study medication. Three criteria defined executive MCI as follows: (1) Montreal Cognitive Assessment (MoCA) score ⩽26; (2) a performance lower than 10th percentile on at least one of the screening tests of executive function—Trail Making Test (TMT) Part B, modified Stroop Interference Test,^15^ Digit Span Backward (DSB) and Sequencing,^16^ and Verbal Fluency;^17^ (3) Functional Activities Questionnaire (FAQ) ⩽7.^18^ Participants were excluded if they had a dementia diagnosis, intolerance to an ACEI or ARB, SBP >200 mm Hg or DBP >110 mm Hg, baseline serum creatinine >1.99 mg/dL, or serum potassium 5.5 mEq/dL.

The CEDAR trial recruited individuals aged ⩾50 years without hypertension who met the National Institute on Aging and Alzheimer’s Association (NIA-AA) diagnostic criteria^19^ for prodromal AD based on: (1) subjective memory concern; (2) MoCA <26, Clinical Dementia Rating (CDR) memory score ⩽0.5,^20,21^ impaired logical memory on the education-adjusted delayed recall of the Wechsler Memory Scale–Revised,^22^ preserved independent activities of daily living on the FAQ; (3) evidence of AD biomarkers positivity. Participants were excluded if they had an intolerance to or current use of ACEI or ARB, a diagnosis of hypertension, baseline serum creatinine >1.99 mg/dL, or serum potassium >5.5 mEq/dL.

### MRI acquisition, processing, and measures

MRI acquisition and analyses were done using a standard protocol and were supervised by study investigators. Sixty-minute MRI scans were performed using a 3T scanner (Magnetom Prisma; Siemens, Erlangen, Germany) at baseline and 12 months. Three-dimensional (3D) T1-weighted images were acquired using a rapid gradient-echo imaging sequence: 170 measurements with repetition time (TR) = 2500 ms (7 min record); voxel size = 3.0 × 3.0 × 3.0 mm; field of view = 220 mm; 48 transversal slices with thickness = 3.0 mm; echo time (TE) = 27 ms; flip angle = 90 degrees. Statistical parametric mapping was used to perform image realignment and Gaussian spatial smoothing.^23^ The images were co-registered with the mean of resting state images, and then normalized to the Montreal Neurological Institute (MNI) template. A mask was used to include only voxels corresponding to brain tissue and exclude voxels corresponding to cerebrospinal fluid.

**Figure 2.**
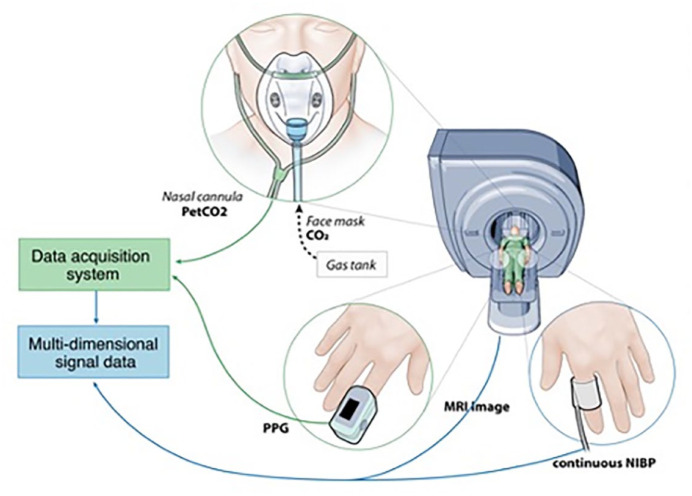
Schematic of the MRI data acquisition system setup. Expired CO_2_ was measured via a nasal cannula, blood pressure using a finger cuff, and PPG using a transducer placed on a finger of the hand opposite that of the pressure cuff.

Respiratory CO_2_ gas was measured using BIOPAC CO_2_ module (CO2100C), BIOPAC Systems hardware (MP 150), and AcqKnowledge^©^ software (4.4.0) and analyzed using MATLAB (R2019a). ETCO_2_ was defined as the value of %CO_2_ signal at the end of a breath and was scaled by 7.13 to convert the units from %CO_2_ to mmHg. ETCO_2_ signals obtained were resampled to match the blood oxygenation level dependent (BOLD) TR (2.5 s), yielding an ETCO_2_ value for each BOLD sample in the time series. Participants underwent a CO_2_ challenge protocol consisting of three phases: (1) a 2-min normocapnic phase where each participant breathed room air, followed by (2) 2 min of exposure to hypercapnia (8% CO_2_ gas) via facemask, and then (3) another 2 min of normocapnia. An illustration of the data acquisition setup is shown in Figure 2.

### Cognitive measures

Neuropsychological testing was performed at baseline, 6-month, and 12-month visits. Episodic memory was assessed using the Hopkins Verbal Learning Test–Revised (HVLT-R) delayed recall and retention. Executive function was assessed using TMT Parts A and B. Part A completion time was subtracted from Part B to correct for motor speed and visuo-perception.^24^ Attention and working memory were assessed using Digit Span Test,^16^ and the Boston Naming Test assessed language.

### Statistical analysis

The primary analysis was performed using individual participant data (IPD) analysis with mixed-effect models described previously.^11^ In both studies, the primary independent variable was the treatment group, and the dependent variable was the percent change in BOLD signal (%ΔBOLD) in response to hypercapnia. Mixed models for repeated measures (MMRM) was used for the analysis, adjusted for the change in ETCO_2_ during hypercapnia (ΔETCO_2_), and sitting SBP (as well as race and pre-study antihypertensive medications for CALIBREX). We conducted IPD meta-analysis, adjusted for study (CALIBREX or CEDAR), and blood pressure. This analysis was to assess if changes in CVR are associated with changes in cognitive performance.

## Results

### Participant characteristics

Of 377 screened for CALIBREX, 176 (47%) were randomized after their antihypertensive medications were tapered: 87 to candesartan and 89 to lisinopril. There were no baseline differences noted including between those who were previously taking ACEI or ARB. Baseline MRI was collected on 72 participants in the candesartan group and 71 participants in the lisinopril group; 55 (76%) participants in the candesartan group and 47 (66%) participants in the lisinopril group underwent a follow-up 12-month MRI scan.

We screened 242 individuals for CEDAR, and 77 (32%) were randomized: 37 to candesartan and 39 to placebo. Baseline MRI was collected on 35 participants in the candesartan group and 36 participants in the placebo group. At 12 months, 31 (89%) participants in the candesartan group and 28 (78%) participants in the placebo group underwent a follow-up MRI.

All participants in both studies tolerated the CO_2_ challenge well, with no discomfort or side effects reported in either study. Study enrollment, drop-out, and group allocations are shown in Figure 1, and the baseline characteristics are provided in Table 1.

**Table 1.** Baseline clinical and demographic characteristics of the CEDAR (N = 59) and CALIBREX (N = 102) trials.

	CEDAR			CALIBREX		
	Candesartan (n = 31)	Placebo (n = 28)	p-value	Candesartan (n = 55)	Lisinopril (n = 47)	p-value
Age (years), mean (SD)	66.0 (8.7)	68.0 (7.9)	0.369	64.5 (7.4)	66.1 (6.7)	0.261
Female, n (%)	12 (38.7)	7 (25)	0.397	24 (43.6)	22 (46.8)	0.903
African American, n (%)	6 (19.4)	6 (21.4)	1	35 (63.6)	29 (61.7)	1
Years of education, mean (SD)	15.8 (3.3)	15.3 (2.1)	0.472	14.8 (2.7)	15.6 (2.6)	0.136
Body mass index	25.4 (5.4)	25.9 (5.2)	0.74	31.6 (6.0)	31.7 (6.7)	0.91
Blood pressure, mean (SD)						
Sitting SBP (mm Hg)	124.0 (13.0)	126.2 (14.4)	0.543	140.6 (20.6)	143.3 (15.2)	0.465
Sitting DBP (mm Hg)	68.3 (9.8)	69.2 (10.3)	0.724	86.0 (12.3)	85.5 (11.6)	0.854
Hypertension, n (%)	0 (0)	0 (0)	1	54 (98.2)	46 (97.9)	1
Received pre-study antihypertensive drug, n (%)^a^	0 (0)	0 (0)	1	39 (83)	43 (78.2)	0.72
Diabetes mellitus, n (%)	0 (0)	1 (3.6)	0.959	17 (30.9)	16 (34)	0.841
Atrial fibrillation, n (%)	3 (9.7)	3 (10.7)	1	7 (12.7)	12 (25.5)	0.161
Coronary heart disease, n (%)	1 (3.2)	2 (7.1)	0.928	6 (10.9)	5 (10.6)	1
Stroke, n (%)	0 (0)	1 (3.6)	0.959	3 (5.5)	5 (10.6)	0.548
Depression, n (%)	6 (19.4)	10 (35.7)	0.263	14 (25.5)	11 (23.4)	0.993
APOE4 positivity, n (%)	24 (77.4)	19 (67.9)	0.595	14 (25.5)	18 (38.3)	0.25
Cognitive scores, mean (SD)						
MoCA	21.1 (3.7)	20.9 (2.8)	0.787	21.3 (3.8)	21.8 (3.5)	0.473
TMT Part B-A	99.0 (89.6)	93.0 (74.6)	0.782	112.1 (81.4)	89.0 (73.3)	0.138
HVLT-R delayed recall	5.1 (4.6)	6.0 (4.0)	0.446	6.5 (3.4)	7.1 (3.2)	0.38
Boston naming test	13.7 (1.4)	13.2 (2.2)	0.302	13.0 (2.0)	13.8 (1.5)	0.022
Digit span forward	9.3 (2.3)	8.4 (2.1)	0.114	8.9 (2.1)	9.0 (2.0)	0.676
Digit span backward	5.8 (2.8)	5.5 (3.0)	0.686	5.3 (2.1)	5.4 (2.0)	0.712

CEDAR: Candesartan’s Effects on Alzheimer’s Disease and Related Biomarkers; CALIBREX: Candesartan versus Lisinopril Effects on the Brain and Endothelial Function in Executive MCI; SBP: systolic blood pressure; DBP: diastolic blood pressure; MoCA: Montreal Cognitive Assessment; APOE4: apolipoprotein E; TMT: Trail Making Test; HVLT-R: Hopkins Verbal Learning Test–Revised.

a Of the total participants on candesartan (n = 86), 31 (36%) participants had no prior diagnosis of hypertension nor were taking blood pressure medication before study enrollment.

### Blood pressure

In the CALIBREX trial, baseline and 12-month sitting SBP were similar between the candesartan and lisinopril groups (baseline mean (standard deviation (SD)) (mm Hg) = 140.63 (20.64), 143.3 (15.19), p = 0.47; 12-month mean (SD) (mm Hg) = 134.04 (18.11), 131.47 (18.17), p = 0.48). In the CEDAR trial, there was no significant baseline difference in sitting SBP, but by 12 months, sitting SBP was lower in the candesartan group compared to placebo (baseline mean (mmHg) = 124.03 (12.98), 126.21 (14.4), p = 0.54; 12-month mean (mmHg) = 116.52 (16.42), 125.52 (15.35), p = 0.03). Supplemental Table S1 shows blood pressure comparisons between treatment groups.

### Voxel-wise and region-of-interest CVR

**Figure 3.**
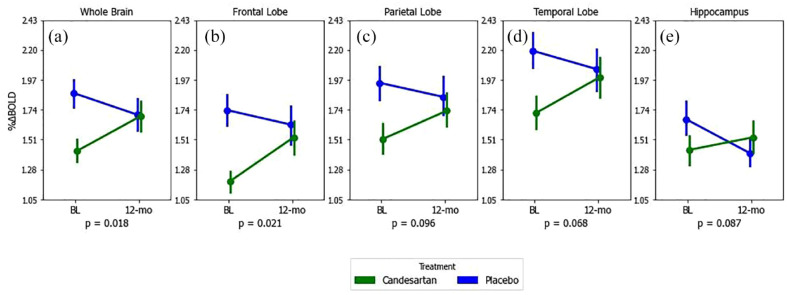
Twelve-month mean CVR changes in candesartan (green) versus placebo (blue) for whole brain (A), frontal (B), parietal (C), temporal (D) lobes, and hippocampus (E)—results from CEDAR trial only. p-values correspond to between-group differences (visit × group), calculated from mixed-model repeated measures of CVR over the study period. Error bars are standard errors. Change in ETCO_2_ during CO_2_ exposure and sitting systolic blood pressure were included as covariates in all repeated measure models.

**Figure 4.**
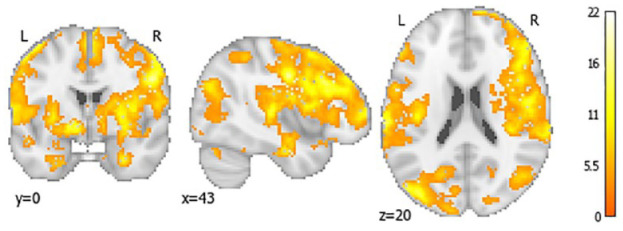
Voxel-wise mapping of significant 12-month CVR changes in candesartan compared to placebo in CEDAR trial only. This voxel-wise color map corresponds to the value of the F statistic with a false discovery rate <0.2.

**Figure 5.**
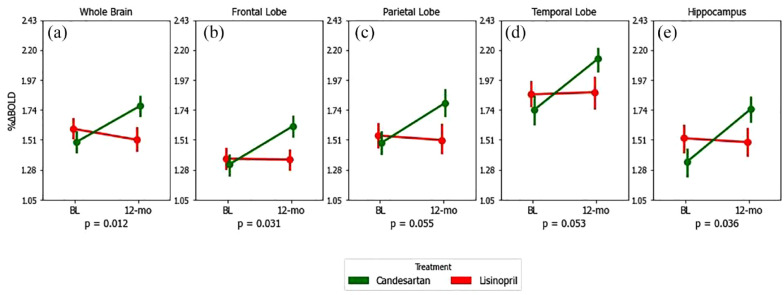
Twelve-month mean CVR changes in candesartan (green) versus lisinopril (red) for whole brain (A), frontal (B), parietal (C), temporal (D) lobes, and hippocampus (E)—results from CALIBREX trial only. p-values correspond to between-group differences (visit × group), calculated from mixed-model repeated measures of CVR over the study period. Error bars are standard errors. Change in ETCO_2_ during CO_2_ exposure, sitting systolic blood pressure, and pre-study antihypertensive medication were included as covariates in all repeated measure models.

**Figure 6.**
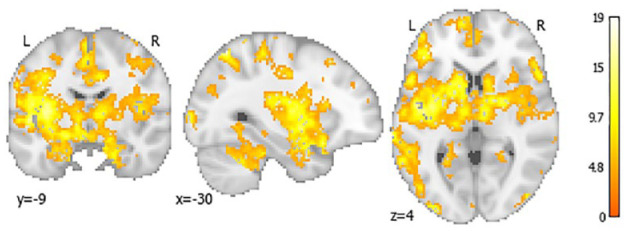
Voxel-wise mapping of significant 12-month CVR changes in candesartan compared to placebo in CALIBREX trial only. This voxel-wise color map corresponds to the value of the F statistic with a false discovery rate <0.2.

In CALIBREX, the whole brain CVR improved significantly in candesartan by 0.28 (95% CI = 0.10, 0.46) and declined in the lisinopril group by −0.08 (95% CI = −0.31, 0.14) (between group p = 0.012). Significant treatment effects were observed in the frontal and hippocampal regions. Similarly in CEDAR, whole brain CVR in candesartan significantly improved by 0.27 (95% CI = 0.006, 0.53) and declined in the placebo group by −0.17 (95% CI = 0.42, 0.08) (between group p = 0.018). There was a symmetrically patterned treatment effect, with most significant voxels located in the frontal, parietal, and hippocampal regions. The voxel-wise maps and lobar group comparisons of 1-year CVR change are shown in Figures 3 to 6.

### Cognitive outcomes

In the CALIBREX trial, candesartan was associated with improved performance in TMT Part B (between-group mean differences = −12.0 (95% CI = −21.7, −2.3) s; p = 0.01) and with improved HVLT-R delayed recall = 0.4 (95% CI = 0.02, 0.8; p = 0.04) compared to lisinopril. In the CEDAR trial, candesartan treatment was also associated with improvement in TMT Part B performance (mean difference = −11.41 s, 95% CI = −11.94, −10.89, p = 0.03), but not with HVLT-R.

To enhance our power to detect underlying mechanisms for the cognitive impacts of candesartan, we performed an exploratory IPD meta-analysis. Candesartan improved CVR in the hippocampus relative to lisinopril and placebo and tended to be associated with improved TMT (B-A) change (slope (standard error (SE)) in candesartan group = −12.5 (7.52), p = 0.087). It was significantly associated with Digit Span Backwards change (candesartan slope (SE) = 0.48 (0.23), p = 0.044). None of these associations were significant in the lisinopril or placebo groups (Supplemental Figures S1 to S4).

## Discussion

This report shows that candesartan was associated with improved CVR compared to lisinopril and placebo. These effects were consistent in the whole brain, as well as across key brain region of interest (ROI) related to cognition—the frontal, parietal, temporal, and hippocampal regions. Further exploratory analyses suggest that improved CVR with candesartan in the hippocampal region may be associated with improved cognition. A few drugs have been shown to affect cerebral vasoreactivity.^25,26^ Prior work has shown that ARB treatment preserves cerebral hemodynamics in humans with stroke or small vessel disease.^27,28^ To our knowledge, this is the first study to show evidence of candesartan improving CVR in two independent populations of MCI.

ARBs block AT1 leading to the likely activation of AT2 receptors in the brain.^29^ AT1 activation increases oxidative stress, neuroinflammation, endothelial dysfunction, and reduced cerebral perfusion.^30^ AT2 activation produces anti-inflammatory effects that can be neuroprotective,^31^ including inhibition of cerebral ischemia.^32^ Previous studies report that ARBs can reduce cerebrovascular inflammation resulting in neuroprotective effects. A randomized study of losartan versus atenolol showed improvements in episodic memory in losartan compared to no change with atenolol (β-blocker) in just 6 months.^33^ Similar benefits to episodic memory have also been reported in studies comparing valsartan with enalapril (ACEI) with valsartan showing more specific vasodilation and endothelial modulation.^34^

MRI-derived CVR maps allow for the examination of regional effects beyond what is seen using transcranial Doppler measurements, previously the most common method for assessing CO_2_ responses. Our data suggest that the effects of candesartan are evident in brain regions that are critical for cognition in both VCI and prodromal AD. These results in the context of brain RAS suggest that ARBs preserve cerebral microvascular function by selective AT1 blockade and AT2 activation.^35^ Decreased CVR has been linked with age-related cognitive decline, AD, and vascular dementia.^36–38^ The observed effect of candesartan on CVR in this study and on cognitive performance in previous studies by our group led us to explore the association of changes in CVR with changes in cognitive performance.^11,14^ Our data suggest that candesartan, compared to other antihypertensives, has a specific effect on CVR and hence may offer a therapeutic modality for cognitive impairment due to the two most prevalent underlying causes—VCI and prodromal AD. Our analysis of the individual trial data and combined data set provides preliminary evidence of replication in two independent populations.

In addition, our exploratory analysis suggests that improved CVR within the hippocampal region may be related to cognitive improvement observed with candesartan. Our findings of specific associations between executive function and working memory cognitive domains in the hippocampal region are not unprecedented. One study has proposed that a prefrontal–hippocampal pathway is important for maintaining executive function.^39^ As such, the specific relationship between hippocampal CVR and executive function or working memory is of great interest for future research.

The strengths of this study include a relatively high generalizability due to the greater representation of African American participants compared to prior studies, and the inclusion of two common MCI types (VCI and prodromal AD) in clinical practice. Also, the detailed measurements of CVR and cognitive battery offer greater depth of understanding of drug effects on CVR. To our knowledge, this is one of the largest clinical trial samples to systematically explore effects of antihypertensive classes that modulate RAS on CVR. The use of BOLD as a surrogate for cerebral blood flow limits this study. However, BOLD CVR is increasingly being applied in many stroke and cognitive research studies.

The ability of ARBs to improve CVR could hold translational potential in cognitively impaired patients with VCI and prodromal AD to prevent further cognitive decline. Future work with a larger sample and an alternative measure of cerebral perfusion would help to further support the use of ARBs in these high-risk groups.

## Supplemental Material

sj-docx-1-wso-10.1177_17474930231153313 – Supplemental material for Effects of candesartan on cerebral microvascular function in mild cognitive impairment: Results of two clinical trials Supplemental material, sj-docx-1-wso-10.1177_17474930231153313 for Effects of candesartan on cerebral microvascular function in mild cognitive impairment: Results of two clinical trials by Brandon Henley, Maureen Okafor, Ambar Kulshreshtha, Antoine Trammell and Ihab Hajjar in International Journal of Stroke
