# Supplementary material for: Effects of candesartan on cerebral microvascular function in mild cognitive impairment: Results of two clinical trials
Source: Int J Stroke. 2023 Jan 30;18(6):736–44. doi: 10.1177/17474930231153313 (PMC10311938; doi:10.1177/17474930231153313)
Supplement: sj-docx-1-wso-10.1177_17474930231153313 – Supplemental material for Effects of candesartan on cerebral microvascular function in mild cognitive impairment: Results of two clinical trials [file sj-docx-1-wso-10.1177_17474930231153313.docx]

**SUPPLEMENTAL METHODS AND RESULTS**

**Interventions**

Enrolled CALIBREX participants were randomly allocated using a 1:1 ratio stratified by race and number of pre-study antihypertensive therapy to either candesartan or lisinopril treatment groups for 12 months. Participants were evaluated every two weeks to achieve a target sitting mean BP of <140/90 mmHg. The initial daily doses of candesartan and lisinopril were 8mg and 10mg, respectively, followed by candesartan titrations to 16mg and 32mg, and lisinopril titrations to 20mg and 40mg. Open-label antihypertensive therapy was added if BP was still >140/90 mmHg at the highest study treatment dose, starting with hydrochlorothiazide at 12.5 mg to 25 mg, amlodipine 2.5 mg to 10 mg, and metoprolol XL 12.5 mg to 25 mg to 50 mg, until the desired BP target was achieved, or all classes were used. Participants did not receive any BP management from other sources throughout the study.

Enrolled CEDAR participants were randomly allocated using a 1:1 ratio to either candesartan (intervention) or placebo (control) treatment groups for 12 months and stratified by use of cholinesterase inhibitors or memantine (taking vs. not taking). All participants were commenced on oral candesartan 8 mg or matched placebo once daily, formulated into identical capsules, with as-needed dose escalations (candesartan 8 mg→16 mg→32 mg or matched placebo) every two weeks until maximum dose was achieved. If a participant became symptomatic, treatment was de-escalated to a tolerable dose.

Cognitive measures of both trials were collected by the same trained study team at baseline, 6 months, and 12 months. Safety measures were monitored at all visits by standard laboratory tests, adverse event reporting, and blood pressure measurements.

**Voxel-wise analysis**

Voxel-wise analysis was performed in MATLAB using Multivariate and Repeated Measures for Neuroimaging toolbox.^1^ Voxel-wise CVR maps were created using the *plot_stat_map* tool in the Nilearn module in Python. These results are presented as the value of the F statistic at a given voxel. ROI analysis was performed by averaging %ΔBOLD values for all voxels corresponding to a given ROI, including whole brain, frontal, parietal and temporal lobes, and hippocampus. MRI data was included as whole brain or lobar BOLD. Adjustment for multiple testing was addressed by in the regional analysis using a Bonferroni correction for 5 regions (whole brain, frontal, parietal and temporal lobes, and hippocampus; <0.01). In the voxel-wise analysis, SPM12 was used to identify voxels and regions that had significant effect by drug treatment with a false discovery rate (FDR) correction at 0.2.^2^

**Adverse Events**

Cough was reported more often among those who received lisinopril (candesartan: 1 [1.2%], lisinopril: 8 [17.0%], placebo: 0). Skin rash was reported more often among those who received candesartan (candesartan: 4 [4.7%], lisinopril: 1 [2.1%], placebo: 0). These results did not unblind the study allocation since all groups reported these adverse events.

**Exploratory metanalytical approach and associations with Cognitive Function**

In an exploratory analysis we used meta-analytical methodologies combining both trials to enhance power and explore effects of CVR changes on cognitive outcomes. These results showed that candesartan was associated with improved CVR relative to lisinopril and placebo. Adjusted mean difference in candesartan significantly improved by 0.27 (95% CI: 0.42, 0.13) vs. decline in the lisinopril group by -0.08 (95% CI: 0.14, -0.31) and by -0.17 (95% CI: 0.08, -0.42) in the placebo group (p-value for between-group differences=0.002). Regionally, all lobes showed similar effects where candesartan therapy associated with improved CVR, while lisinopril and placebo showed declining CVR at 12 months. Supplementary figures S1 and S2 show results of a meta-analysis of the two studies comparing CVR effects between the three groups.

We further explored the association between CVR change and change in the key cognitive measures over the study period. The association between CVR change (δBOLD) and TMT (B-A) change within each of the three treatment groups was marginally significant in the candesartan group alone (slope (SE) = -12.5 (7.52), p=0.087). The association between CVR change (δBOLD) and DSB change showed significant improvement in the candesartan group (slope (SE) = 0.48 (0.23), p=0.044), but not in the lisinopril or placebo groups. These exploratory findings are presented in the online Supplement (Figures S3 and S4).

**SUPPLEMENTAL TABLE**

**Table S1.** Mean (SD) sitting systolic blood pressure at baseline, 6 months, and 12 months in the candesartan, lisinopril, and placebo treatment groups (N=161).

|  | **Baseline**  **Mean (SD)** | **6 months**  **Mean (SD)** | **12 months**  **Mean (SD)** | ***P-value***  ***(group*** x ***visit)*** |
| --- | --- | --- | --- | --- |
| Candesartan (n=86) | 134.7 (19.9) | 123.5 (17.7) | 127.7 (19.4) | *0.095* |
| Lisinopril (n=47) | 143.3 (15.2) | 127.6 (12.1) | 131.5 (18.2) |  |
| Placebo (n=28) | 126.2 (14.4) | 121.4 (16.4) | 125.5 (15.4) |  |

Abbreviations: BL, baseline; 6-mo, 6 months; 12-mo, 12 months.

The reported p-value corresponds to the effect of the interaction of the treatment group and study visit (group x visit) obtained from the following model: SBP = Visit + Group + Visit × Group + Study, where “visit” is treated as a continuous variable (1=BL, 2=6-mo, 3=12-mo).

**SUPPLEMENTAL FIGURES**


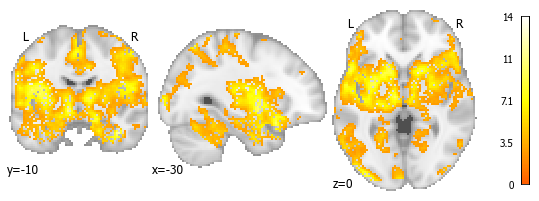


**Figure S1.** Voxel-wise mapping showing significant improvement in cerebrovascular reactivity (CVR) from baseline to 12 months in candesartan compared to lisinopril and placebo. This voxel-wise colormap reflects the F statistics (drug x visit) from the voxel-wise analysis after FDR correction.


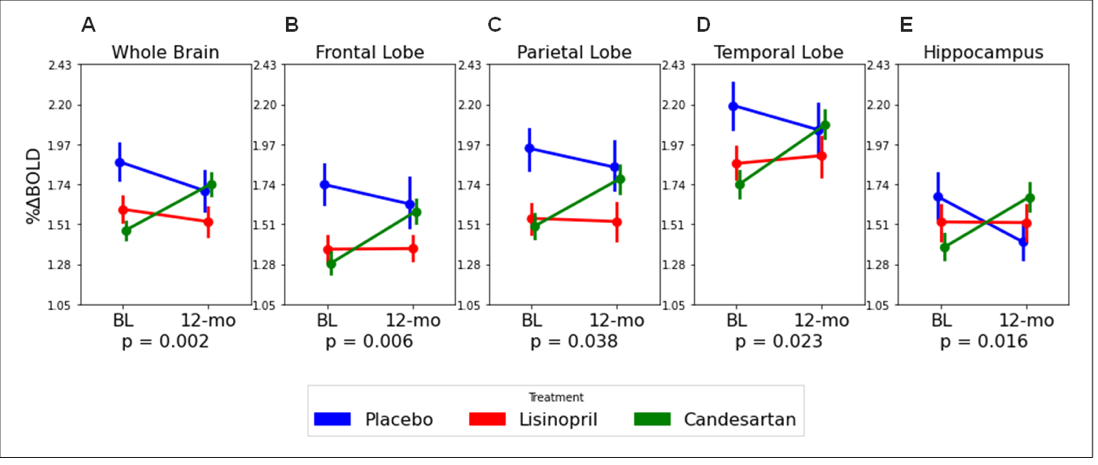


**Figure S2.** Mean CVR at baseline and 12 months by treatment group: candesartan (green), lisinopril (red), and placebo (blue) on the change in CVR from baseline to 12 months in whole brain (A), frontal (B), parietal (C), temporal (D) lobes, and hippocampus (E). The p-values correspond to the between-group differences ($visit\times group$), calculated from mixed model repeated measures of CVR over the study period and adjusted for ΔETCO_2_, race, sitting SBP, number of pre-study antihypertensive medications, and study (MCI type). Error bars are standard errors.


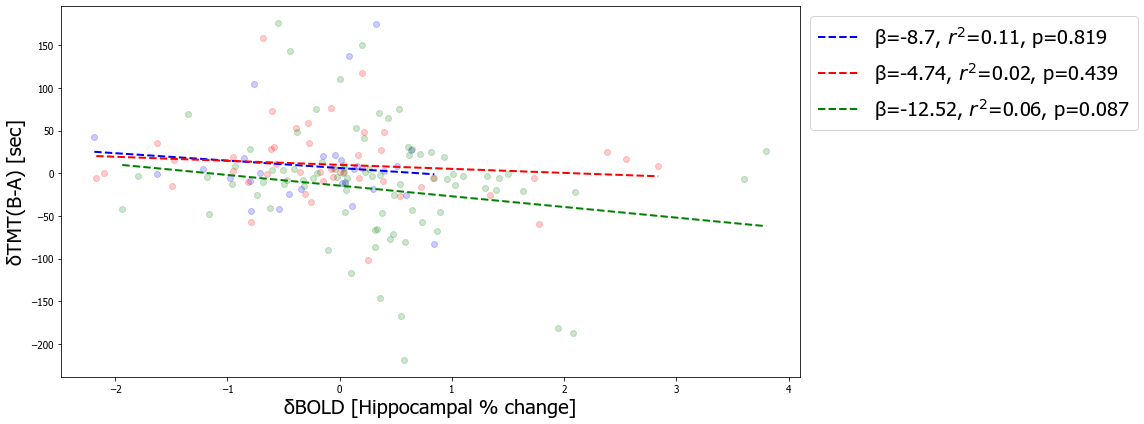


**Figure S3.** Scatter plot of the association between δTMT Part B-A and δBOLD in the hippocampus for the combined trial data. The association within the candesartan group was marginal (green dashed line, p=0.087) but not significant in the lisinopril (red dashed line) or placebo (blue dashed line) groups. β=slope. Abbreviations: TMT, Trail Making Test; BOLD, blood oxygenated level dependent imaging.


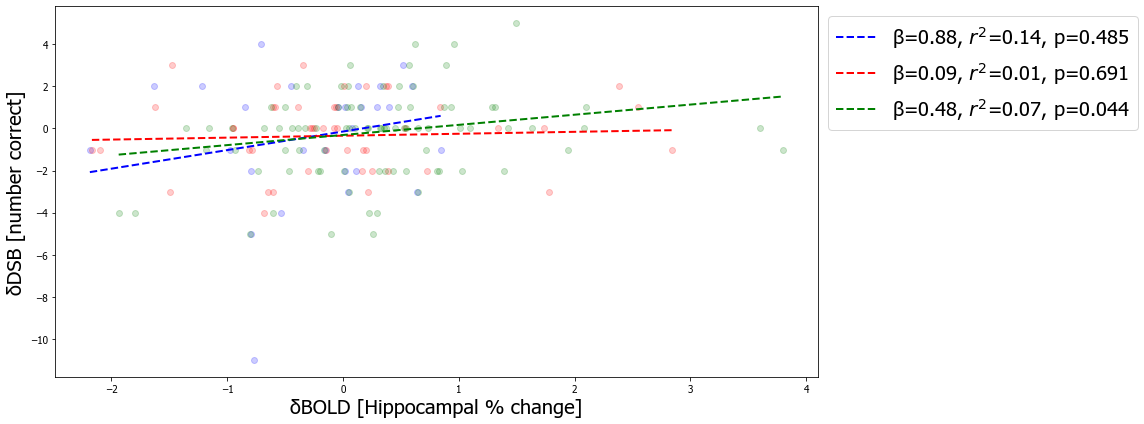


**Figure S4.** Scatter plot of the association between δDSB and δBOLD in the hippocampus for the combined trial data. The association within the candesartan group was significant (green dashed line, p=0.044) but not significant in the lisinopril (red dashed line) or placebo (blue dashed line) groups. β=slope. Abbreviations: DSB, digit span backward test; BOLD, blood oxygenated level dependent imaging.

References

1. McFarquhar M, McKie S, Emsley R, et al. Multivariate and repeated measures (MRM): A new toolbox for dependent and multimodal group-level neuroimaging data. *Neuroimage* 2016; 132: 373-389. 20160224. DOI: 10.1016/j.neuroimage.2016.02.053.

2. Nichols T and Hayasaka S. Controlling the familywise error rate in functional neuroimaging: a comparative review. *Stat Methods Med Res* 2003; 12: 419-446. DOI: 10.1191/0962280203sm341ra.
